# Supplementary material for: Identification and Characterization of Tomato SWI3-Like Proteins: Overexpression of SlSWIC Increases the Leaf Size in Transgenic Arabidopsis
Source: Int J Mol Sci. 2019 Oct 16;20(20):5121. doi: 10.3390/ijms20205121 (PMC6829904; doi:10.3390/ijms20205121)
Supplement: Supplementary file 1 [file ijms-20-05121-s001.docx]

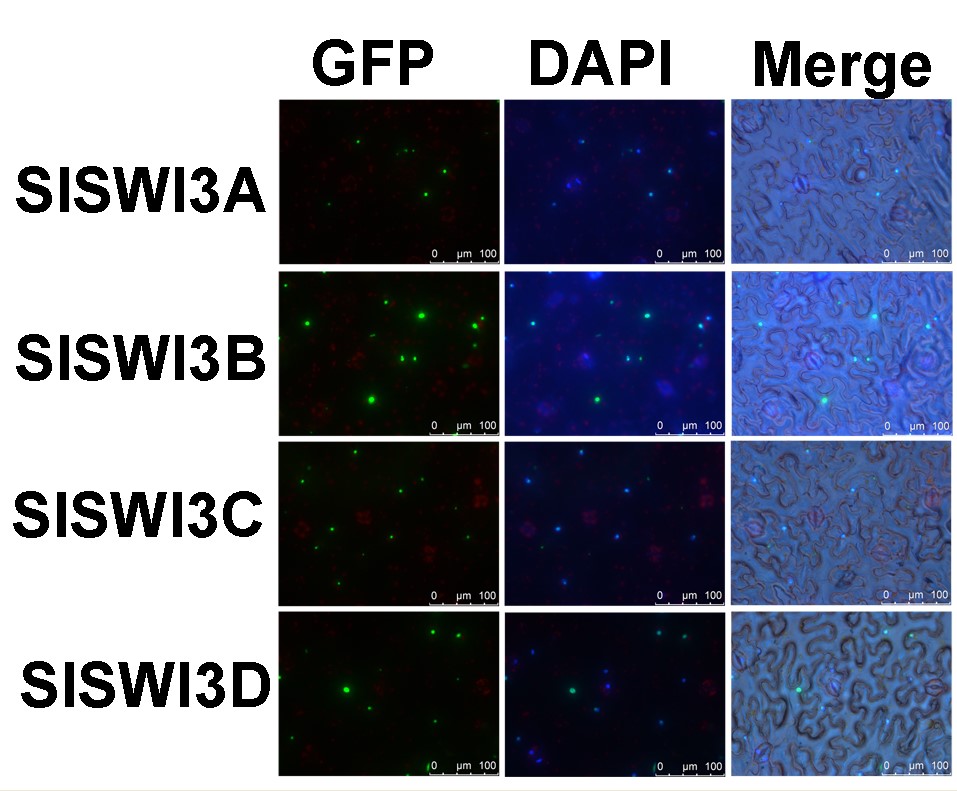


**Figure S1.** The subcellular localization of tomato SWI3-like proteins. From left to right: the GFP, DAPI fluorescence spectrum and overlay signals. The scale bar corresponds to 100 μm.


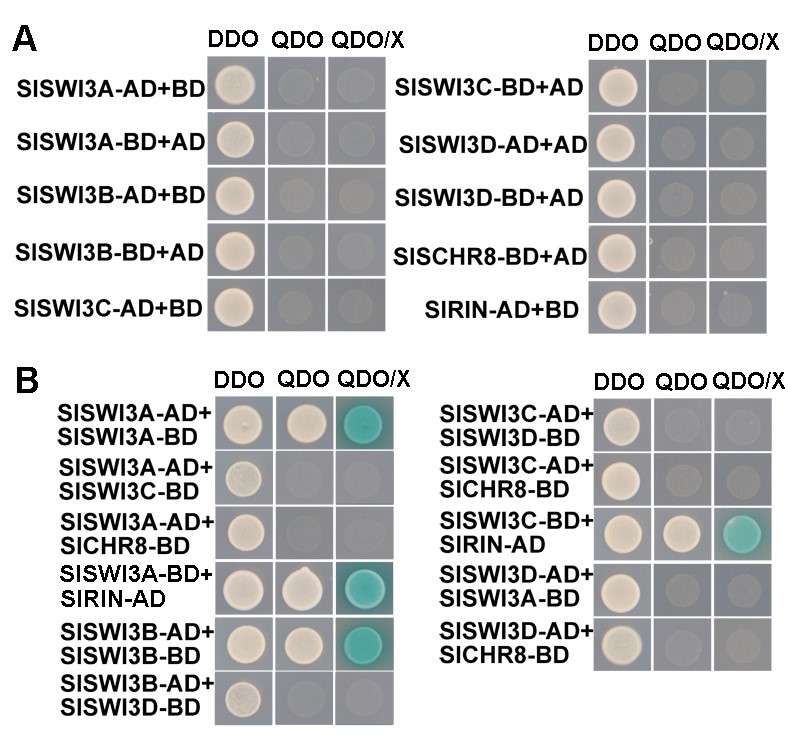


**Figure S2.** Self-activation (**A**) and the interactions of SlSWI3s with each other (**B**) were examined in yeast cells.


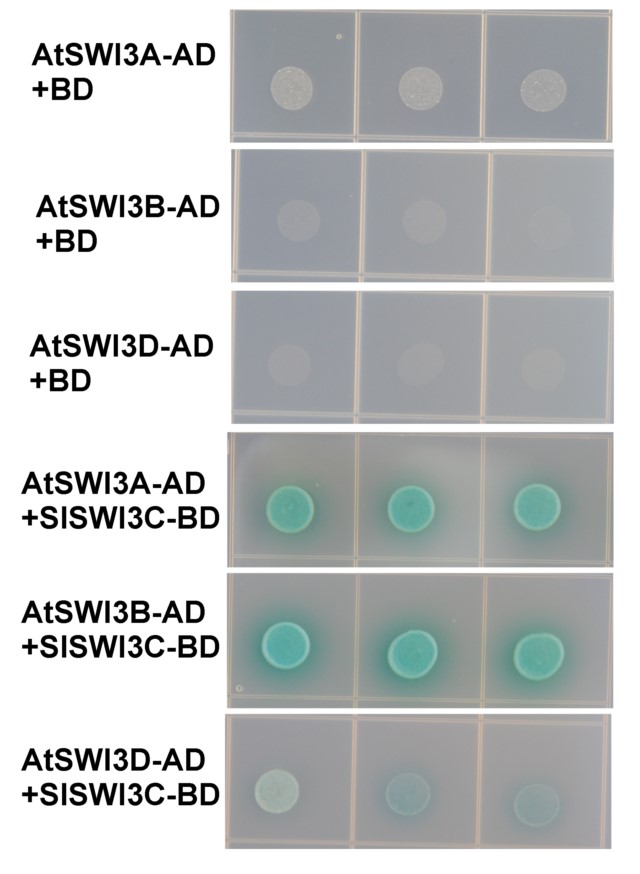


**Figure S3.** The interactions of SlSWI3s with AtSWI3s were examined in yeast cells.


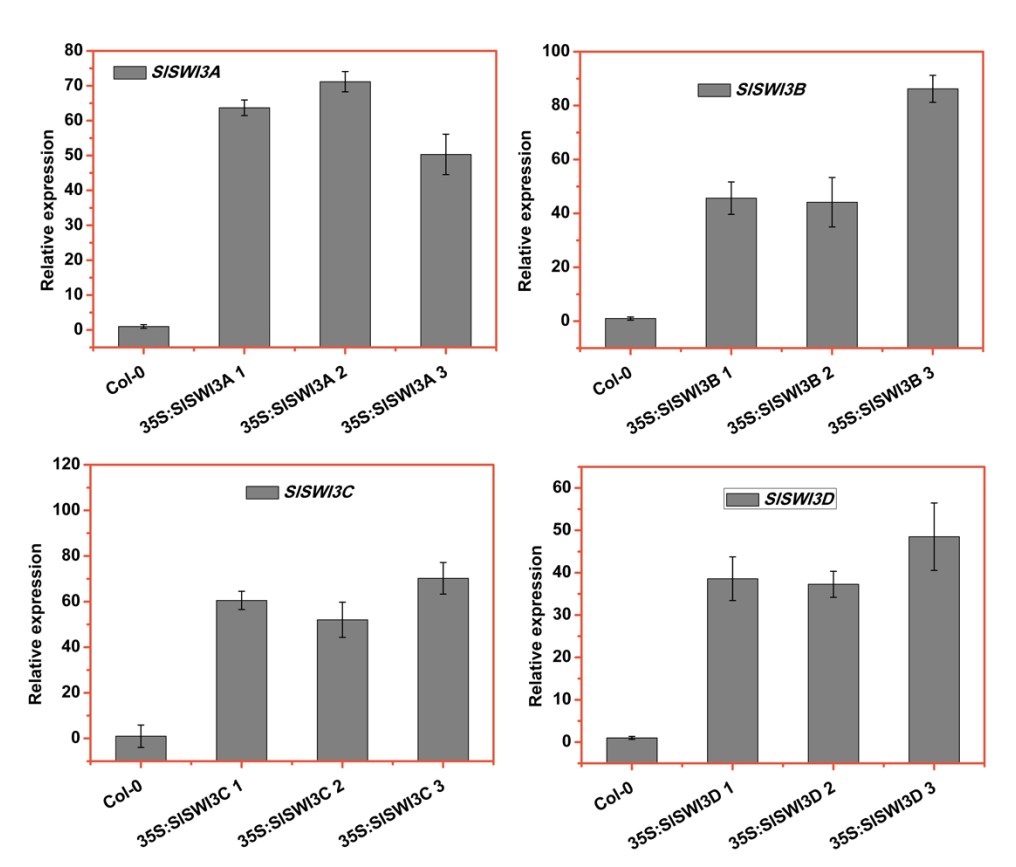


**Figure S4.** The transcripts of *SlSWI3s* in 21-day-old transgenic *Arabidopsis* seedlings.


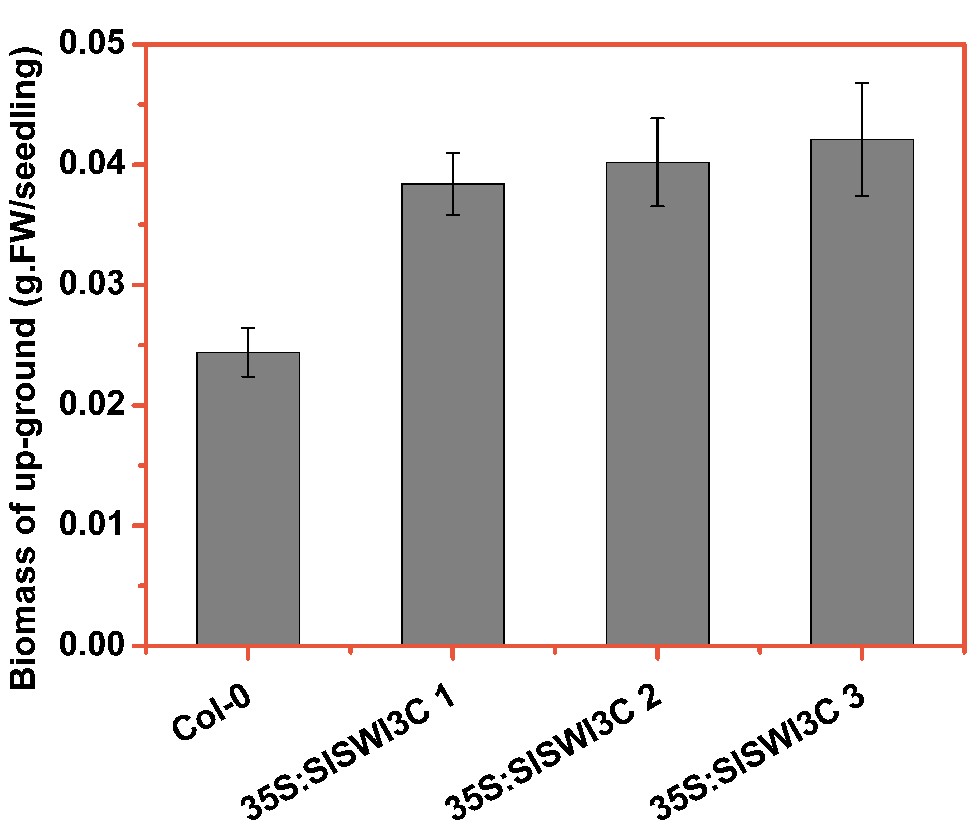


**Figure S5.** The fresh weights (FW) of up-ground parts of Col-0 and *SlSWI3C* overexpressing plants grown in soil for two weeks.


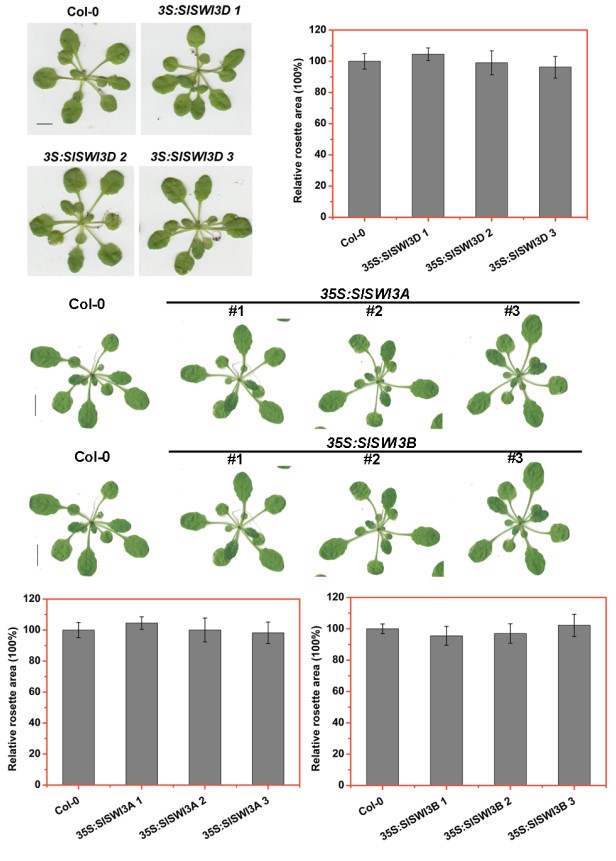


**Figure S6.** The phenotype of overexpression of *SlSWI3A*, *SlSWI3B,* and *SlSWI3D* transgenic *Arabidopsis* seedlings.

**Table S1.** Primers used in this study.

| **Primer for qPCR** | **Forward** | **Reverse** |
| --- | --- | --- |
| *GRF6* | CCTCAAGAAAGCCTCCTCCTA | CAGAGCAGCTTTTGATTGACC |
| *GRF3* | TGGTAGTGAGGAAGTGGTTGG | TCGTATGGCCTCTTGTTGTTC |
| *GRF5* | CATGGTCTCTGGTGTTCCTGT | GCTCTGGATCTGGTTTTCTCC |
| *HB33* | TTGCAAGTCATCAGAAAGAGGA | TGAATGTTGTTCCGAATGAGAC |
| *SlSWI3C* | CAGCAGGAGATGGAAGATGAA | CACTAATCCGGTCCCTACTTTC |
| *SlSWI3A* | GTCGTTGGTGGGTGATATAAGT | TTCCGCTGGAGCATGAATAG |
| *SlSWI3B* | TTCAGTCACCGATCCCAAAG | CCAACGGGAATAACTGGGAATA |
| *SlSWI3D* | GGAGGATTTGGAGGCATTAGAG | AGAGAACCAACCAGCATGAG |
| *SlATIN* | AGGCAGGATTTGCTGGTGATGATGCT | ATACGCATCCTTCTGTCCCATTCCGA |
| *AtACTIN2* | CTAAGCTCTCAAGATCAAAGGC | AACATTGCAAAGAGTTTCAAGG |
| **Primer for constructs** |  |  |
| SlRIN-AD | ATGGAGGCCAGTGAATTC  ATGGGTAGAGGGAAAGTAGAA | TCATCTGCAGCTCGAGC  AAGCATCCATCCAGGTACAAC |
| SlCHR8-AD | ATGGAGGCCAGTGAATTC  ATGGTGGCTCAGATAGAGACC | TCATCTGCAGCTCGAGCGG  ATATTAAGCTTGATCTCCTC |
| SlCHR8-BD | CCATGGAGGCCGAATTC  ATGGTGGCTCAGATAGAGACC | GCCGCTGCAGGTCGACG  GGATATTAAGCTTGATCTCCTC |
| SlSWI3C-AD | ATGGAGGCCAGTGAATTC ATGCCAGCTTCCTCCTCAGAGAC | TCATCTGCAGCTCGAGC ACCTAAACCTGTTTTTGTCCC |
| SlSWI3C-BD | CCATGGAGGCCGAATTC ATGCCAGCTTCCTCCTCAGAGAC | GCCGCTGCAGGTCGACG ACCTAAACCTGTTTTTGTCCC |
| SlSWI3D-AD | ATGGAGGCCAGTGAATTC ATGGAGGAGAAACGGAAGGACA | TCATCTGCAGCTCGAGC CTTTGAAGGCTGCATTGAATT |
| SlSWI3D-BD | CCATGGAGGCCGAATTC ATGGAGGAGAAACGGAAGGACA | GCCGCTGCAGGTCGACG  CTTTGAAGGCTGCATTGAATT |
| SlSWI3A-AD | ATGGAGGCCAGTGAATTC  ATGGATGCATCTCGCTTGTA | TCATCTGCAGCTCGAGCT  ATGCTGCTGGTGCTGCTTT |
| SlSWI3A-BD | CCATGGAGGCCGAATTC  ATGGATGCATCTCGCTTGTA | GCCGCTGCAGGTCGACGT  ATGCTGCTGGTGCTGCTTT |
| SlSWI3B-AD | ATGGAGGCCAGTGAATTC  ATGCCCGAATCAGGCGAAAT | TCATCTGCAGCTCGAGCTTC  TGCCTTAACATCTATCA |
| SlSWI3B-BD | CCATGGAGGCCGAATTC  ATGCCCGAATCAGGCGAAAT | GCCGCTGCAGGTCGACGTT  CTGCCTTAACATCTATCA |
| SlSWI3A-pEAQ-GFP | GCCCAAATTCGCGACCGGT  ATGGATGCATCTCGCTTGTA | CTTTGCTAGTCATACCGGTTATG  CTGCTGGTGCTGCTTT |
| SlSWI3B-pEAQ-GFP | GCCCAAATTCGCGACCGGT  ATGCCCGAATCAGGCGAAAT | CTTTGCTAGTCATACCGGTT  TCTGCCTTAACATCTATCA |
| SlSWI3C-pEAQ-GFP | GCCCAAATTCGCGACCGGT  ATGCCAGCTTCCTCCTCAGAGAC | CTTTGCTAGTCATACCGGTACCT  AAACCTGTTTTTGTCCCAGA |
| SlSWI3D-pEAQ-GFP | GCCCAAATTCGCGACCGGT  ATGGAGGAGAAACGGAAGGACA | CTTTGCTAGTCATACCGGTCTT  TGAAGGCTGCATTGAATT |
| SlRIN-pEAQ-GFP | GCCCAAATTCGCGACCGGT  ATGGGTAGAGGGAAAGTAGAA | CTTTGCTAGTCATACCGGTA  AGCATCCATCCAGGTACAAC |
| SlCHR8-Nluc | GGGACGAGCTCGGTACC  ATGGTGGCTCAGATAGAGAC | Tacgagatctggtcgac  GGATATTAAGCTTGATCTCC |
| SlSWI3B- Nluc | GGGACGAGCTCGGTACC  ATGCCCGAATCAGGCGAAAT | Tacgagatctggtcgacttc  TGCCTTAACATCTATCA |
| SlSWI3B-Cluc | CGTCCCGGGGCGGTACC  ATGCCCGAATCAGGCGAAAT | Aagctctgcaggtcgac  TTCTGCCTTAACATCTATCA |
| SlSWI3A- Nluc | GGGACGAGCTCGGTACC  ATGGATGCATCTCGCTTGTA | tACGAGATCTGGTCGAC  TATGCTGCTGGTGCTGCTTT |
| SlSWI3C- Nluc | GGGACGAGCTCGGTACC  ATGCCAGCTTCCTCCTCAGAGAC | Tacgagatctggtcgacacc  TAAACCTGTTTTTGTCCC |
| SlSRIN-Cluc | CGTCCCGGGGCGGTACC  ATGGGTAGAGGGAAAGTAGA | Aagctctgcaggtcgac  AGCATCCATCCAGGTACAAC |
| SlSWI3C-pHB | TCTCAAGCTTGGATCC ATGCCAGCTTCCTCCTCAGAGAC | CGTCACTAGTGGATCC  ACCTAAACCTGTTTTTGTCCCAGA |
| SlSWI3A-pHB | TCTCTCAAGCTTGGATCC  ATGGATGCATCTCGCTTGTATA | ACCGTCACTAGTGGATCC  TATGCTGCTGGTGCTGCTTTGA |
| SlSWI3B-pHB | TCTCTCAAGCTTGGATCC ATGCCCGAATCAGGCGAAATCA | ACCGTCACTAGTGGATCC TTCTGCCTTAACATCTATCACC |
| SlSWI3D-pHB | TCTCTCAAGCTTGGATCC ATGGAGGAGAAACGGAAGGACAC | ACCGTCACTAGTGGATCC CTTTGAAGGCTGCATTGAATTTC |
